# Development of a Disposable Polyacrylamide Hydrogel-Based Semipermeable Membrane for Micro Ag/AgCl Reference Electrode

**DOI:** 10.3390/s23052510

**Published:** 2023-02-24

**Authors:** Eivydas Andriukonis, Marius Butkevicius, Povilas Simonis, Arunas Ramanavicius

**Affiliations:** 1 State Research Institute Center for Physical and Technological Sciences, Sauletekio Ave. 3, 10257 Vilnius, Lithuania; 2 Department of Bioanalysis, Institute of Biochemistry, Life Sciences Center, Vilnius University, Sauletekio Ave. 7, 10257 Vilnius, Lithuania; 3 Department of Physical Chemistry, Faculty of Chemistry and Geosciences, Vilnius University, Naugarduko Str. 24, 03225 Vilnius, Lithuania

**Keywords:** Ag/AgCl reference electrode, chrono amperometry, cyclic voltammetry, electrochemistry, three electrode cell, polyacrylamide hydrogel, semipermeable membrane, ion diffusion

## Abstract

Currently, Ag/AgCl-based reference electrodes are used in most electrochemical biosensors and other bioelectrochemical devices. However, standard reference electrodes are rather large and do not always fit within electrochemical cells designed for the determination of analytes in low-volume aliquots. Therefore, various designs and improvements in reference electrodes are critical for the future development of electrochemical biosensors and other bioelectrochemical devices. In this study, we explain a procedure to apply common laboratory polyacrylamide hydrogel in a semipermeable junction membrane between the Ag/AgCl reference electrode and the electrochemical cell. During this research, we have created disposable, easily scalable, and reproducible membranes suitable for the design of reference electrodes. Thus, we came up with castable semipermeable membranes for reference electrodes. Performed experiments highlighted the most suitable gel formation conditions to achieve optimal porosity. Here, Cl^−^ ion diffusion through the designed polymeric junctions was evaluated. The designed reference electrode was also tested in a three-electrode flow system. The results show that home-built electrodes can compete with commercial products due to low reference electrode potential deviation (~3 mV), long shelf-life (up to six months), good stability, low cost, and disposability. The results show a high response rate, which makes in-house formed polyacrylamide gel junctions good membrane alternatives in the design of reference electrodes, especially for these applications where high-intensity dyes or toxic compounds are used and therefore disposable electrodes are required.

## 1. Introduction

Electrochemistry is rapidly expanding toward the development of electrochemical sensors and some other bioelectronics-based devices [1,2]. Most of these electrochemical devices are based on electrochemical systems with three electrodes, where one electrode serves as a reference [3]. Currently in electrochemistry, one of the most popular and widely used reference electrodes (RE) is the silver/silver chloride (Ag/AgCl) electrode [4,5]. Ag/AgCl reference electrodes are widespread and applied due to stable, potential, durable, and simple construction. The Ag/AgCl reference electrodes consist of silver wire thoroughly coated with an AgCl layer and immersed in a KCl solution with some kind of semipermeable ion-selective barrier [6,7]. Because of their simple construction, micro-sized Ag/AgCl reference electrodes can be easily constructed in the laboratory. The main problems related to the design of micro reference electrodes are the electrode choice of the body and the ion-selective barrier. Today, porous glass membranes are most widely used in the design of commercial electrodes because they are chemically inert, and their pore sizes can vary from 1 nm to 1 µm [8]. However, manipulating glass structures is quite challenging because they require high temperatures [9]. Another option is based on the application of polymeric membranes [10]. Polymeric membranes are convenient because various membrane shapes can be designed, and the porosity of the polymer can be easily customized; therefore, they are used in the formation of selective ion membranes [11,12,13]. Due to these properties, hydrogels could be applied as semipermeable membranes.

Hydrogels, also referred to as aqua gels, are a series of “soft” and “wet” materials that have highly porous, three-dimensional, cross-linked polymer networks consisting of hydrophilic polymers that allow them to retain large amounts of water without significantly compromising their structure. Due to distinctive and characteristic properties--biodegradability, biocompatibility, hydrophilicity, softness, flexibility and fluffiness--hydrogels can be used in various areas, including agriculture [14], drug delivery [15], tissue engineering, regenerative medicines [16], biosensors, and other bioelectronics-based devices [17,18,19]. The properties of hydrogels can also be improved by various structural modifications, namely increased freezing temperature [20], increased electrical conductivity [21], capacitance [22], and self-healing, a property in high demand [17,21,22,23]. Polyacrylic acid or its derivative polyacrylamide (PAM) are basic laboratory polymers and are often chosen as the matrix base for more complex copolymer hydrogel formation. These polymers are highly stretchable and easily chemically modifiable as a result of an abundance of functional groups. Due to these remarkable properties, we have applied PAM gel as a membrane for reference electrodes in our present research.

Previously, simple reference electrodes were reported using agar as semipermeable material using 3D printing to make reference electrodes [24,25]. In one study, authors report reference electrodes with a working area of 7 × 7.5 mm and electrode body size of 11 × 11 × 6 mm [24], while another study reported electrodes with a working area of 0.5625 mm^2^ × π while its body was 9 mm^2^ × π [25].

In the case of leak-proof Ag/AgCl reference electrodes, the pore size of the polymeric membrane should be comparable to the hydrated potassium or hydrated chloride ion radii, taking into account that K^+^ and Cl^−^ ions are of similar diameter (0.31 and 0.32 nm, respectively, non-hydrated radii 0.14 and 0.18 nm), while the polymer membrane can be as thin as 2 nm [10]. Therefore, polyacrylamide gel (PAM-G) has been used in several reference electrode designs because: (i) this polymer is very commonly available in laboratories; (ii) hydrogel formation is based on easy procedures; (iii) the gel formation process is fast; (iv) gel-based structures are stable over time when hydrated; and (v) the relationship of pore size to the concentration of the monomer used is well-known [12]. In addition, polyacrylamide (PAM) synthesis does not involve the use of volatile solvents.

We developed a fast procedure for the formation of cheap, simple, small-scale, disposable Ag/AgCl reference electrodes using a polyacrylamide (PAM-G) matrix as the electrode semipermeable membrane. The disposable part was based on standard automatic pipette tips and cast membrane plugs. Other parts of this reference electrode, however, can be reused. Polyacrylamide quickly forms gels characterized by predictable and repetitive properties suitable for the design of reference electrodes devoted to electrochemical devices. To promote the concept and novelty, we performed electrochemical comparison experiments of in-house-built reference electrodes with commercially available versions. In addition, the chloride ion flux through 10–60% PAM-G was determined using optical methods. The PAM-G structure was also evaluated by a scanning electron microscope (SEM).

## 2. Materials and Methods

### 2.1. Chemicals and Solutions

For PAM-G preparation and electrochemical measurements, all materials were obtained from Sigma-Aldrich unless otherwise noted. Arylamide, bis-acrylamide, ammonium persulfate, TEMED, potassium chloride, concentrated sulfuric acid (36%), concentrated hydrogen peroxide (32%), sodium phosphate, (hydroxymethyl) ferrocene, and silver wire (99.99%) of two different radii (0.4 and 0.05 mm) were used for the experiments. Plastic disposable pipette tips made of polypropylene were used as bodies for the RE half-cells. Acrylamide and bis-acrylamide monomers were used in a ratio of 37.5 to 1, respectively, for the preparation of water-based bulk polymerization solutions. Bulk polymerization solutions with different monomer concentrations were prepared: 10, 20, 30, 40, 50, and 60% *m*/*v* (further on the acrylamide monomer solution). During this investigation, a 15% *m*/*v* water solution of ammonium persulfate was used. The Piranha solution was freshly prepared prior to polypropylene oxidation. Piranha solutions were prepared from sulfuric acid and hydrogen peroxide in a 3:1 ratio.

### 2.2. The Design of the Electrode

#### 2.2.1. Casting Polyacrylamide Hydrogel Membranes

The PAM-G casting tips of disposable polypropylene pipette tips were oxidized in Piranha (H_2_SO_4_:H_2_O_2_, 3:1) solution for 1–2 h. The oxidation of polypropylene with Piranha introduces polar OH- functional groups as it was reported in some other references [26]. In order to functionalize just a particular part of disposable polypropylene-based pipette, only the tip (10 mm) was immersed in a Piranha solution. After the treatment, the Piranha solution was washed out with a large amount of deionized water (DI) and dried with pressurized air.

The PAM-G casting mixture recipe was modified from those previously used in the preparation of gels in protein polyacrylamide gel electrophoresis [27]. An amount of 1 mL of 10, 20, 30, 40, 50, or 60% acrylamide monomer solution was mixed with 15 µL of 15% ammonium persulfate and 2 µL of TEMED. The reaction mixture was vortexed at maximum speed for 15 s in a table top vortex. Then fixed volumes (2, 4, 8, 12 µL) of PAM-G setting were quickly transferred from the master mix to the preoxidized pipette tips. The transfer was performed with a pipette by ejecting the volume (2, 4, 8, 12 µL) of gel into an unattached pre-oxidized tip. The tips with PAM-G were then left for 10 to 15 min to solidify in air, then were carefully filled, immersed in 3 M KCl, and left to fully cure for at least 1 h.

#### 2.2.2. Electrochemical Formation of the AgCl-Based Layer

Before electrochemical formation of the AgCl-based layer, the silver wire was cleaned with sandpaper and rinsed with acetone to remove any impurities. Then the electrochemical formation of an AgCl-based layer on a silver electrode was achieved by immersing the wire in a solution containing 3 M KCl and passing a current of 0.3 mA/cm^2^ through the electrode for 15–20 min until the electrode was adequately plated. The color of a well-plated wire becomes dark.

### 2.3. Electrochemical Measurements

Electrochemical experiments were performed with Gamry 300 potentiostat (Gamry Instruments, Warminster, PA, USA). All experiments were carried out in a three-electrode glass cell using a 3 mm diameter glassy carbon working electrode (BASI, West Lafayette, IN, USA), a platinum wire as a counter electrode (BASI), our home-built reference electrode, commercial reference electrode (silver chloride) (PalmSens, Houten, The Netherland), or saturated calomel electrode (SCE) (BASI, West Lafayette, IN, USA). Before the experiments, the glassy carbon electrode was polished with a 0.3 μm aluminum oxide slurry, rinsed with deionized water (DI), and sonicated for 4 min in DI and 4 min in acetone to remove non-bound particulates. After sonication, the working electrode was thoroughly washed with DI. All electrochemical measurements were performed at room temperature in a working buffer solution (WBS), pH 7.0, containing 100 mM of phosphate ions. Cyclic voltammograms (CV) were recorded in a 0.1 mM hydroxymethylferrocene (HMF) solution in WBS at a potential sweep rate of 100 mV/s. Some PAM-G membranes were cast into the RE body and left in 3 M KCl at room temperature for six months prior to examination.

In this study, designed reference electrodes were tested in a continuous flow 3-3electrode electrochemical cell. The electrochemical cell was home-built and was suitable for the incorporation of reference electrodes designed here. The cell was 3D-printed in clear resin consisting of a stainless-steel auxiliary electrode and a working platinum electrode. The entire constant flow-through electrochemical cell was assembled with two M4 × 60 mm screws. The approximate working volume of the flow cell was ~120 µL, which was evaluated from the 3D model as the largest distance between the RE and the auxiliary electrode (9.46 mm) and multiplied by the flow cell’s inner diameter surface area (π·4 mm^2^). During the RE testing, the flow cell was connected to a calibrated peristaltic pump, and the signal stability was evaluated at various flow rates. The flow rate was turned on and off. Between the “turn on” cycles, the flow rate increased by 5 mL/min. The next “turn on” cycle was performed before the registered signal had increased significantly. A solution of 5 mM potassium ferrocyanide and 0.1 M sodium phosphate (pH 7.0) was used for the evaluation of the performance of our designed RE. Measurement was performed at a 0.4 V potential set to the working electrode. The increase in current was measured during the “turn on” cycles when a fresh potassium ferrocyanide solution was pumped into the flow cell and the redox process occurred at the working electrode. Measurements were made until the physical limit of the peristaltic pump was reached, a flow rate of 55 mL/min. Almost all lower flow rate signals were stable. Only after reaching 45 mL/min did the signal start to flocculate and also reach the amplitude of signal “saturation”.

### 2.4. Membrane Permeability Measurements

The permeability of the PAM-G membrane was determined by creating osmotic pressure on both sides of the membrane. The pipette tip with cast PAM-G was filled with 200 µL of 3 M KCl, immersed in 75 mL of DI, and left for 20–24 h. Subsequently, a 2 mL sample was taken from 75 mL of DI mixed with 30 µL of 1 M AgNO_3_. The translucency of the sample was then evaluated with a Dynamica Halo RB-10 spectrophotometer (United Kingdom). The results were compared with those determined in samples of known KCl concentration.

### 2.5. Sample Preparation for SEM

Various PAM-G samples were frozen and then lyophilized overnight. Prior SEM imaging samples were thermally vacuum-deposited with a conductive chrome layer.

## 3. Results and Discussion

### 3.1. Assessment of Permeability of PAM-G-Based Membrane

**Figure 1:**
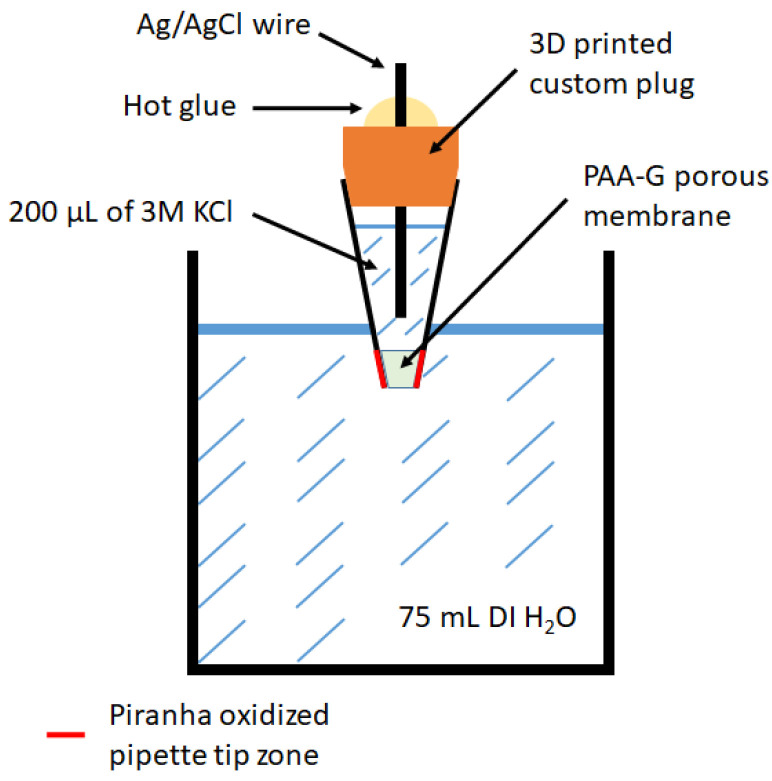
Scheme representing the design of Ag/AgCl-based reference electrode and setup for the permeability tests. Detailed construction of Ag/AgCl-based reference electrode is described in Section 2.2; detailed description of permeability tests is presented in Section 2.4.

During the preparation of reference electrodes, the formation of a semi-permeable layer within the capillary is more advantageous than the attachment of semipermeable membranes to the tip of this capillary. In the design phase, 200 µL pipette tips were chosen as the RE body because they were just the right size for handling and have convenient volume, a sufficiently thin end, and, most importantly, a conical shape. The conical shape is one of the requirements as it can be easily press-fitted into electrochemical prototype devices while ensuring a leak-proof fit while still being easily replaceable. Even though the construction of this type of reference electrode was described more than 20 years ago [7], the use of a glued-on top membrane was inappropriate because it is difficult or nearly impossible to create small circular membranes. Furthermore, the application of commercial Naf ion membranes is also problematic because the choice of membranes with a pore size less than 1 µm is limited even though it is much larger than those of porous glass membranes (2–4 nm). To leave the electrode size defined by the pipette tip, it was decided to make a membrane inside of the pipette tip; thus, casting of the uncured polymer solution was the best option as it can potentially provide better adhesion to the electrode body. In Figure 1, the design of the reference electrode is presented. The oxidation of the pipette tips was implied during the testing phase because, prior to oxidation, they showed inconsistent permeability of the ions. During oxidation with Piranha solution, the polypropylene surface was presumably covered with functional -OH groups [26] which, in our case, were observed to promote the adhesion of PAM-G adhesion to the pipette tip surface.

Membrane permeability experiments revealed that PAM-G membranes were permeable at a moderate diffusion rate (Table 1). Thus, the results show that approximately 0.06 M of Cl^−^ diffuses over the 20 h experiment time. Taking into account the diameter of the pipette tip opening, which was 0.4 mm, the average diffusion rate was estimated at 1.6 × 10^3^ mol × m^−2^ × s. Some correlation was observed between PAM concentration and the amount of diffused Cl^−^. The increased concentration of PAM in a solution used for PAM-G formation decreases PAM-G-based membrane permeability, which implies that the density of the PAM in hydrogel structure increases and the size of pores filled by solvent decreases. On the basis of membrane thickness, in this experiment, no correlation was observed between the different volumes used in membrane preparation and diffused Cl^−^ ions. From a practical standpoint, however, 8 µL membranes performed the best and were still easily produced manually.

### 3.2. Assessment of PAM-G Structure and Porosity

**Figure 2:**
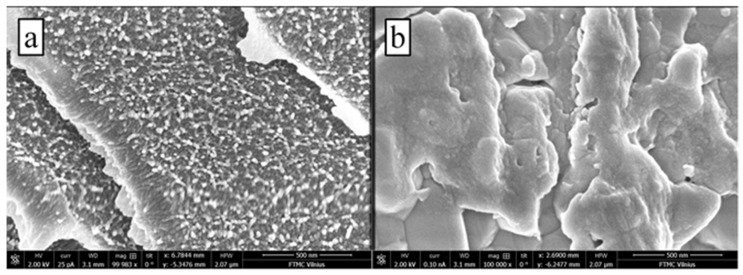
SEM images of (**a**) PAM-G and (**b**) PAM-G cast with 1 M KCl. Both gels were cast from 40% PAM concentration solution. Both images were taken at 10^5^ magnification. PAM-G were made up from nano sized (~15 nm) spherical structures, while PAM-G + KCl from monolithic folds.

The gel structures were also inspected with scanning electron microscopy. Two different types of membranes were prepared: (i) with 1 M KCl in the gelation solution, and (ii) without any KCl in the gelation solution. It was hypothesized that the gel formed from a gelation solution containing KCl would have some advanced network of microstructures and nanostructures. Before imaging, the PAM-G samples were frozen and lyophilized. It was determined that the lyophilization disrupted the gel and that large voids appeared in the hydrogel samples. We believe, however, that the formed micro and nanostructures were not affected by this treatment. On evaluation, the addition of KCl alters swelling and the formation of PAM-G gels [28]. Gel swelling in the presence of salts is a common phenomenon and is also documented with other types of polymers, such as polyvinyl alcohol [29], etc. In our study, gels formed from KCl containing gelation solution were observed to have microstructures that resemble rarely porous odd-shaped monolithic folds (Figure 2b), while gels without KCl were porous and contained consistent nanosized spheres that form the whole microstructure (Figure 2a). Similar PAM-G structures have also been described by other authors [30,31]. The PAM-G structure represented in Figure 2a was more regular and uniform, which also led us to believe that the integrity and performance of the whole structure is more unified. Thus, PAM-G membranes formed from gelation solution without any KCl can potentially be more efficient in the sense of controlling the semipermeability of formed gels. Hence, reported PAM-G-based reference electrodes are suited for short-term measurements. During long-term measurements, however, the KCl from the inner solution tends to diffuse slowly through the pores of the hydrogel, so a slight potential drift can be observed during long-lasting electrochemical experiments.

### 3.3. Characterization of Ag/AgCl Electrodes

**Figure 3:**
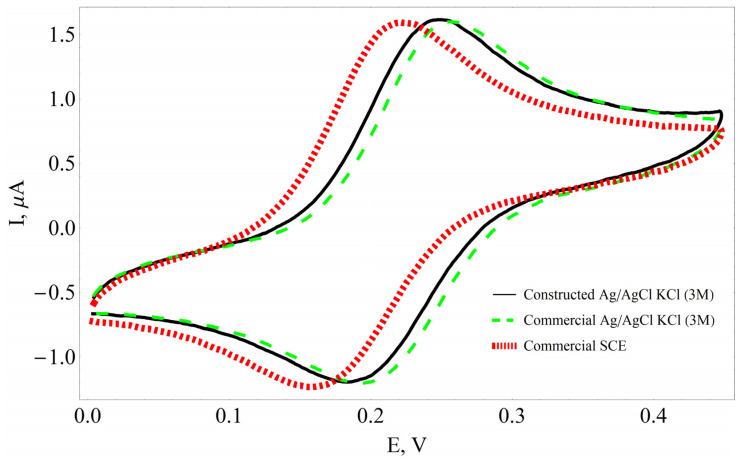
Cyclic voltammograms recorded using different types of reference electrodes. CV recorded in 0.1 mM hydroxymethylferrocene (HMF) in WBS, pH 7.0. The potential sweep rate is 100 mV/s.

Cyclic voltammetry was performed to assess and compare the performance of RE reported here with that of commercially available reference electrodes (SCE and Ag/AgCl electrode). The redox couple of HMF_(ox.)_/HMF_(red.)_ based on the oxidized/reduced states of the ions (Fe^2+^/Fe^3+^) was examined by cyclic voltammetry in a three-electrode electrochemical setup. A good correlation was observed between CVs with the homemade electrode and the commercially available reference electrodes (Figure 3). The difference between the homemade electrode and the commercially available Ag/AgCl-based reference electrode may have occurred as a result of experimental error. According to the literature, the difference between Ag/AgCl in 3 M KCl and SCE is approximately 32 mV [32,33], but our measured potential difference between these electrodes determined during multiple independent measurements was 29 ± 2 mV. The peak potential difference and peak height remained constant in CVs after a multiple number of potential cycles, which is an indication of the stability of the reference electrode designed during this research. Furthermore, the thickness and composition of the membrane do not affect the position and shape of the CVs.

**Figure 4:**
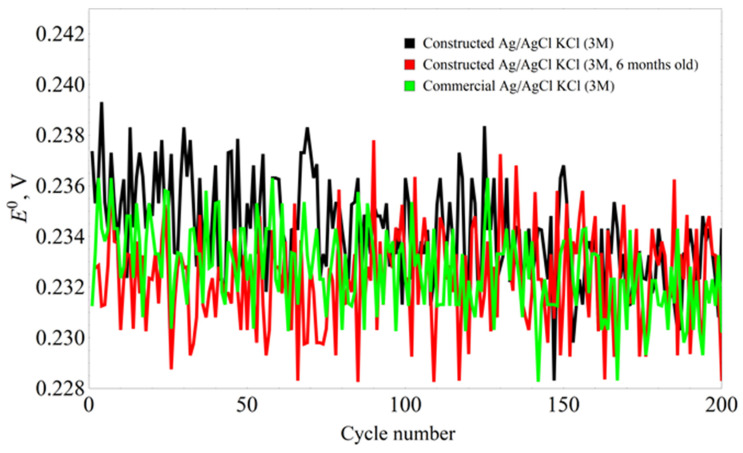
Assessment of redox potential stability during long lasting cyclic voltammetry-based experiment. Two hundred potential cycles were performed. During experiments, freshly made reference electrodes and those of six months of storage were compared with commercial reference electrodes. CV recorded in 0.1 mM hydroxymethylferrocene (HMF), potential sweep rate–100 mV/s.

CV measurements were performed to test the stability of the reference electrode designed during this research. Initially, the stability of the RE potential was tested in an extended cyclic voltammetry experiment. Here, designed REs were used in cyclic voltammetry-based measurements by 200 voltammetry cycles (Figure 4); during this experiment, no significant shift of redox-active compound (HMF) reduction potential was observed. The same REs were similarly evaluated after six months of shelf life. During that time, electrode bodies with PAM-G membranes were suspended in 3 M KCl. Before use, the REs were washed and filled with a fresh 3 M KCl solution, and an Ag/AgCl electrode was placed into the cell. After cyclic voltammetry measurements, no significant difference in performance was observed once again, suggesting that PAM-G-based membranes do not disintegrate over at least six months and that their age does not affect the accuracy of measurements. It should be noted that the preparation of PAM-G based membranes is very simple in comparison to more sophisticated hydrogel-based structures used in electrochemical systems [34,35]. Thus, PAM-G based membranes can be prepared in batches and kept until use, making them an excellent candidate for the design of disposable electrodes.

**Figure 5:**
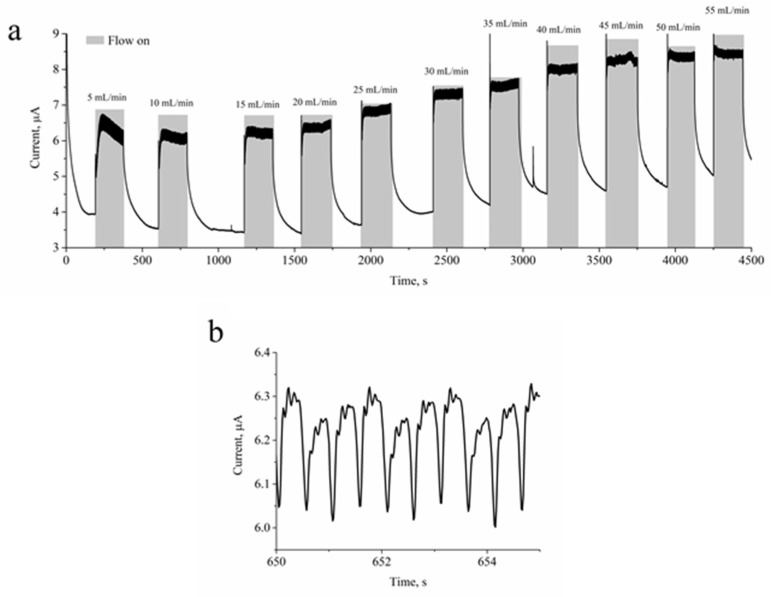
(**a**) Three-electrode configuration flow cell experiment where RE electrode was tested at various flow rate conditions. Measurements performed using 5 mM potassium ferrocyanide and 0.1 M sodium phosphate buffer, pH 7.0, at 0.4 V set to working platinum electrode. (**b**) High resolution graph of the same experiment at flow rate of 10 mL/min from 650 to 655 s.

We performed experiments in a flow-through cell and determined that our created testing system was able to perform well when the flow rate was below 35 mL/min. Therefore, the designed reference electrode performs well under various flow-rate conditions. With high sampling values set for the potentiostat, it was also observed that the setup system was able to pick up the variation of the current induced by peristaltic pump pulsation (Figure 5b). This finding indicated that the electrode system designed here is characterized by fast response times and helps to visualize small variations of concentration. Furthermore, it indicates that, during a single flux of the solution induced by the peristaltic pump, there is enough time for the redox reaction to increase the concentration of ferrocyanide significantly, also resulting in a decrease of measured current.

## 4. Conclusions

PAM-G-based reference Ag/AgCl electrodes were designed. Polyacrylamide has been reported as suitable material for the development of a semipermeable membrane for a reference Ag/AgCl electrode due to good reproducibility and limited chloride ion diffusion. It was demonstrated that the diffusion rate of chloride ions is mostly determined by the hydrogel porosity. Such reference electrodes based on PAM-G can be easily fabricated in great numbers using common laboratory materials; therefore, PAM-G is a viable material for the design of a disposable reference electrode. During this research, PAM-G-based semipermeable membranes show good electrochemical properties compared to commercial reference electrodes, as well as stable potential over long-term experiments. Reported PAM-G-based reference electrodes are suitable for long-term measurements because the KCl from the inner solution tends to diffuse slowly through the pores of the hydrogel, while the lowest amount of diffused chloride ions was 1.8% over 20 h. The proposed membrane casting methodology can be adapted for different types of reference electrodes based on a semipermeable or ion-selective membranes and could be easily integrated into the body of electrochemical devices.

## Figures and Tables

**Table 1:** Percentile fraction of diffused Cl^−^ through the membranes of various sizes and concentrations after 20 h. The results are shown with standard deviation, n = 6.

	The Volume of PAM-G Used to Cast Membrane, µL		
PAM-G Concentration, %	4	8	12
10	4.8 ± 2.5	2.4 ± 1.9	5.0 ± 1.0
20	3.7 ± 0.5	2.2 ± 0.9	3.2 ± 0.6
30	3.4 ± 1.0	2.2 ± 0.4	1.8 ± 0.5
40	2.1 ± 0.4	1.8 ± 0.1	1.9 ± 0.3
50	2.3 ± 0.7	1.9 ± 0.3	2.1 ± 0.4
60	2.4 ± 0.5	2.1 ± 0.2	1.9 ± 0.3

## Data Availability

Data will be available upon request.
